# A Novel Distributed Vibration Sensor Based on Fading Noise Reduction in Multi-Mode Fiber

**DOI:** 10.3390/s22208028

**Published:** 2022-10-20

**Authors:** Lidong Lu, Xingchen Su, Chenglong Zhang, Qinghao Gao, Hongwei Yang

**Affiliations:** School of Electrical and Information Engineering, Anhui University of Technology, Maanshan 243002, China

**Keywords:** optical fiber sensor, vibration location, multi-mode fiber

## Abstract

Multi-mode fiber (MMF) is used in a polarization-sensitive optical time domain reflectometer (OTDR) for vibration event location and spectrum analysis. The vibration events acting on MMF are considered to be the optical polarization state and phase diversifying process for fading noise reduction. In addition, data averaging with continuous positions and the fast Fourier transform (FFT) method is proposed to extract the spectrum of the vibration events. In the experiment, the vibration events are loaded at the positions of 5.167 and 10.145 km, respectively, along MMF. The experimental results demonstrate that the vibration event can effectively diversify the optical polarization state and phase of the Rayleigh scattering light to make the averaged OTDR trace behind the vibration position converge rapidly, which helps to locate corresponding vibration events and extract the vibration spectrum. It is inferred that the new distributed vibration sensor shall have a lower false alarm rate, as it can greatly reduce the errors caused by randomness of the sensing light signals. Additionally, it also saves time in comparison with the method that analyzes the vibration spectra for all the positions along the fiber under test.

## 1. Introduction

Optical fiber is passive and lightweight and can be distributed along the object to measure corresponding parameters, such as temperature, strain, vibration, etc. Based on the optical time domain reflecting structure, the light scattering spectrum has been adopted to locate and identify the events occurring along the sensing fiber. The Rayleigh scattering-based distributed optical fiber sensors are widely applied to practical engineering, for example, fault location in optical fiber communication cables [1], leakage monitoring of oil pipelines [2,3], state parameter monitoring in the petroleum industry such as asphaltene [4,5,6], and partial discharge sensing on the power transmission lines [7,8]. The distributed vibration sensing technology has become a hot issue, especially in the power industry. The distributed temperature sensors are applied to the power cable for hot spot detection [9,10,11] and the distributed vibration sensors (DVSs) are used for wind deviation and galloping analysis of optical fiber ground wire (OPGW) [12,13,14,15]. Generally, MMF is adopted in the Raman optical time domain reflectometer (ROTDR) for distributed temperature sensing as it possesses a large mode field diameter, which helps to suppress the nonlinear optical effects in the optical fiber and ensures the sensor obtains a high dynamic range. Nowadays, ROTDR is widely used in power cable tunnels for cable temperature monitoring and fire warning in the tunnel environment. Additionally, DVS such as the polarization-sensitive OTDR and phase-sensitive OTDR have been put into practical use for the external force damage monitoring of power cable tunnels or intrusion detection [16,17,18], and these events often have a low vibration frequency below several hundred Hertz [18]. The vibration events are located by optical time domain reflecting technology and identified by vibration spectrum analysis [19,20,21]. Usually, the vibration event is located by the successive difference or trace subtraction method, and then using the FFT algorithm, the vibration spectrum is extracted [22,23,24]. By successive difference or trace subtraction methods, the vibration position can be quickly located, but it may cause a very high false alarm rate, as the fading noise, especially the randomness of the optical polarization state and phase, can also make the OTDR trace fluctuate [25]. However, this method just locates the vibration events and cannot identify the type of vibration events. So, the extraction of the vibration spectrum for event identification becomes a very important issue, especially for the distributed acoustic sensors (DASs). The FFT method can be used to analyze the spectrum of vibration events and locate corresponding events, but it takes a lot of time to complete the data processing for each position along the sensing fiber [22,26,27], or a digital signal processing (DSP) module with a very fast data computing ability should be adopted in the DVS system, which will undoubtedly increase the cost.

At present, the polarization-sensitive or phase-sensitive OTDRs are generally based on single mode fiber (SMF). However, for both temperature and vibration event monitoring in the power cable tunnel, it is very important to consider the compatibility of the sensing media. Since MMF has been widely laid on power cables, we should consider making most of MMF rather than laying SMF again for vibration event location along the power cable. So, a new DVS scheme that adopts MMF as the sensing media should be studied. Although vibration event location and identification algorithms, such as the successive difference method and fast Fourier transform method, can be used in the MMF-based DVS scheme, corresponding experiments are needed to demonstrate the feasibility. In this paper, the fading noise and the polarization state change caused by the vibration events are both considered to be a random process. Using the data averaging method, the fading noise can be effectively reduced, so that it makes the OTDR trace at the vibrating position and each position behind become smooth, which provides a good way to locate the vibration event. Although the trace average will reduce the dynamic measurement performance of the proposed system, it avoids the randomness of the optical polarization state of the Rayleigh light and can improve the measurement accuracy for vibration events with low frequencies [28]. Then, to extract the spectrum of the vibration event, the data at the vibration position and the positions behind it are also averaged to improve the signal to noise ratio (SNR) of the vibrating signal. At last, the spectra of vibration events are extracted by the FFT algorithm. This indicates that the DVS scheme can be integrated with the commonly used DTS system by improving the optical path structure design; so, the hybrid optical fiber sensing system can simultaneously monitor temperature events and vibration events [29,30].

## 2. Experimental Set-Up

**Figure 1:**
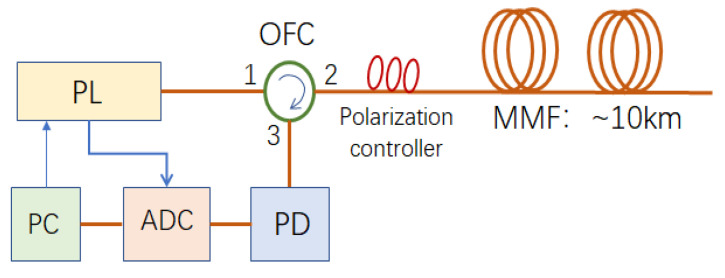
Schematic diagram of the experimental set-up.

The experimental set-up is shown in Figure 1. The pulse laser (PL) generates probe pulse light, and it is launched into the first port of the optical fiber circulator (OFC), and then it is output from the second port of OFC and its polarization state is adjusted by the polarization controller. Then, the probe light pulse comes into the multi-mode fiber (OM1 62.5/125) with a length of about 10 km. The laser linewidth of PL is about 6 MHz. The pulse width is 10 ns and the pulse peak power is about 33 dBm. The backscattered Rayleigh light of the propagating light pulse in the MMF passes through the second and the third ports of OFC and at last it is converted into the voltage signal by the photodetector (PD). The voltage signal output from PD is sampled by the data acquisition card (DAQ) with a sampling rate of 100 Msps. The PL, OFC, polarization controller, and PD are with SMF, so the connection between the polarization controller and MMF may cause light power loss. The loss from the polarization controller to MMF can be omitted, and the measured loss from MMF to the polarization controller is 7.2 dB. DAQ collects the voltage signals and sends the corresponding data to a personal computer (PC), where the data are processed and analyzed and the vibration information along the fiber under test (FUT) is presented. PC also controls PL and sets the parameters of the probe pulse such as the pulse width and peak pulse power.

PL sends the trigger signal to DAQ to synchronize the signal receiving. DAQ has a data average function, and it can continuously collect and save the averaged data. The data sampling period for an arbitrary position along MMF is decided by the product between the round-trip time of the probe pulse and the data averaging number. In the experiment, the pulse period is set to 200 μs and the data average number is 10, so the sampling frequency for the vibration event extraction is 500 Hz. Additionally, by the Nyquist sampling theorem, it can discriminate vibration events with a frequency less than 250 Hz. In addition, an optical fiber coil of about 20 m in length and 10 cm in diameter is set at the positions of 5.167 and 10.145 km, respectively, along MMF. Additionally, vibration events are loaded by rapidly slapping the optical fiber coil with a frequency less than 10 Hz. When the probe pulse propagates in MMF, it will generate backscattering Rayleigh light. As the fading noise, including the coherent Rayleigh noise and polarization noise [28], leads the fluctuation in the OTDR trace, if the vibration event can effectively diversify the phase and polarization state of Rayleigh scattering light, the OTDR trace will be smoothed by the trace averaging. By statistic theory, trace averaging can reduce the fading noise and improve the measurement dynamic range.

In this experiment, DAQ processes the data averaging function, and it can continuously average the data from specified measurement times and save the results to be one OTDR trace data. Then, it starts the next averaging without stopping. The first position where the OTDR trace begins to converge is determined as the position of the vibration event. In one pulse period, the data (trace) collected by DAQ can be written as Equation (1):

(1) $$Tr=R_{i}^{L}(L_{1},L_{2},\cdots ,L_{N})$$

where $R_{i}^{L}$ is the time domain voltage signals collected by DAQ, *L* is the fiber length, $L_{i}$ is the discrete position digitized by DAQ, and the total sampling points (positions) is *N*.

As DAQ has a data averaging function, the trace data output from DAQ in one pulse period can be written as Equation (2):

(2) $$P_{j}=\frac{1}{M}\sum_{i=1}^{M}R_{{}_{i}}^{L}(L_{1},L_{2},\cdots ,L_{N})$$

where *M* is the average number and $P_{j}$ is the averaged result in the *j*th measurement.

To further reduce the fading noise in the OTDR trace, the trace average number can be increased, as shown in Equation (3):

(3) $$P=\frac{1}{K}\sum_{j=1}^{K}P_{j}$$

where *K* is the number of the total traces adopted for the average. To extract the vibration spectrum, the trace data with a certain position range is first averaged, and it is expressed in Equation (4):

(4) $$P_{j}^{uv}=\frac{1}{V-U}\sum_{s=U}^{V}P_{j}(L_{s})$$

where $L_{s}$ represents a certain position, and *U* and *V* are the starting point and ending point, respectively. Then, to obtain the vibration spectrum, an array is obtained, which is the time domain data from continuous measurements and data averaging with a certain position range, as shown in Equation (5). Then, by *FFT*, the vibration spectrum can be extracted by Equation (6):

(5) $$W=[P_{1}^{uv},P_{2}^{uv},\ldots ,P_{K}^{uv}]$$

(6) S=FFT(W)

## 3. Results and Discussion

**Figure 2:**
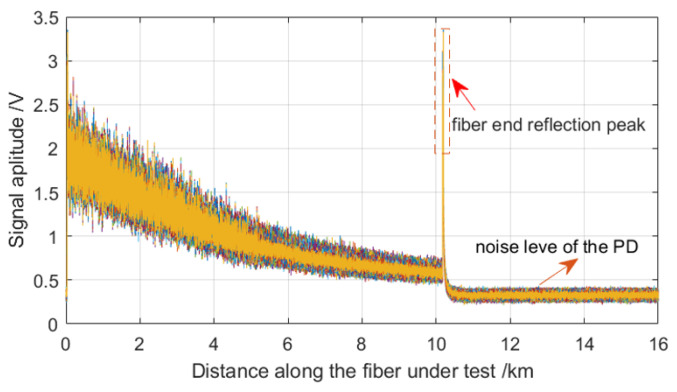
The overlapped 640 OTDR traces.

Without loading the vibration events, the OTDR traces are obtained. Figure 2 overlaps 640 OTDR traces. It can be seen that the OTDR traces have obvious Rayleigh fading noise that makes the OTDR trace fluctuate drastically [1,2,3]. So, we suspect that the vibration events that are loaded on MMF should diversify the optical polarization state and phase of the Rayleigh light so that after using the data averaging method, the OTDR trace becomes smooth [28]. Averaging the OTDR trace will reduce the sampling frequency for vibration event extraction. However, the detection of vibration events with a low frequency does not need a wide frequency response range. In addition, it is also necessary to lower the sampling frequency to collect the vibrating signals within several signal periods for spectrum analysis by FFT.

**Figure 3:**
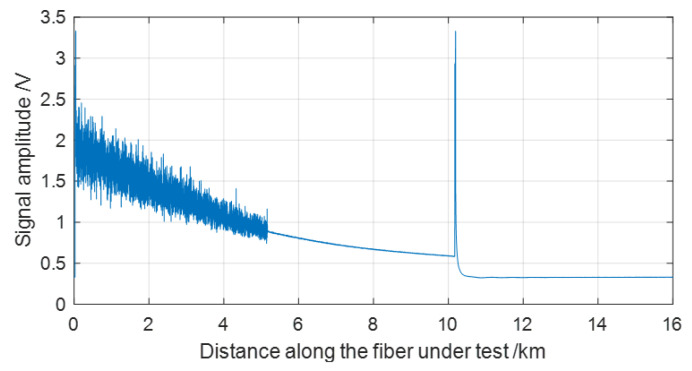
The OTDR trace after data averaging with the vibration event loaded.

Next, by FFT, the vibration spectra can be obtained. If the sampling frequency remains very high, the data volume is relatively large, and it correspondingly increases the data processing time for vibration event analysis [29,30]. For example, if the sampling rate of DAQ is 100 Msps, the signal sampling time is 0.2 ms, the frequency of the vibration signal is 5 Hz, and the sampling process must include at least two signal periods, then the collected data volume is 80 Mbytes for a 16-bit DAQ. So, a DSP module with a fast computing ability is necessary to obtain the vibration spectrum. However, if the OTDR traces collected from each signal sampling period are averaged by 10 times, then the collected data volume is just 8 Mbytes, which is beneficial for fast event extraction. However, averaging the OTDR trace reduces the frequency response range for vibration spectrum analysis because it correspondingly decreases the signal sampling frequency. Therefore, it is essential to balance the data processing ability and vibration spectrum measurement range of the sensing system. In this experiment, the vibration event loaded on MMF is lower than 10 Hz, the OTDR traces are averaged by 10 times, and 640 averaged OTDR traces are obtained for spectrum analysis. The signal sampling period is 0.2 ms. So, it takes 2 ms to obtain one averaged OTDR trace, and the total signal sampling time for the 640 OTDR traces is 1.28 s, which corresponds to at least 6 signal periods if the frequency of the vibration signal is about 5 Hz. Figure 3 shows the OTDR trace obtained by averaging the 640 OTDR traces in Figure 2. It is observed in Figure 3 that the OTDR trace becomes smooth at the vibration position and each position behind, and this effect is extremely obvious. Therefore, it provides a new method for locating vibration events along FUT. Through the comparison of Figure 2 and Figure 3, it is found that by trace averaging, in the final OTDR trace, the fluctuation (by fading noise) in front of the vibrating position is also reduced, but it is not greatly changed. It is assumed that the vibration events diversify the optical polarization state and phase [28], which reduces the fading noise in the OTDR trace within the corresponding region in Figure 3.

**Figure 4:**
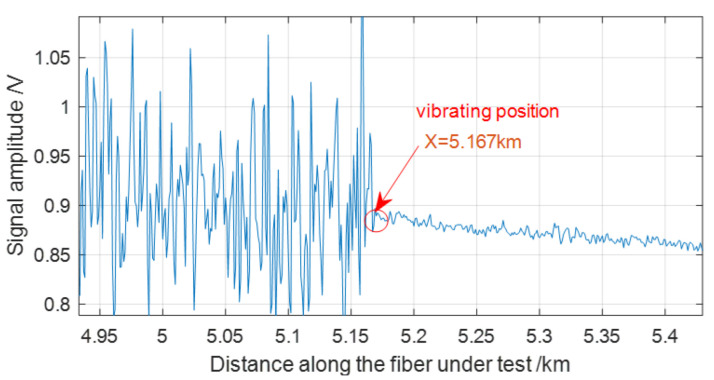
Contrast between the trace fluctuation before and behind the vibration position.

Figure 4 shows the trace details in Figure 3, which indicates that the vibration event is loaded at the position of 5.167 km on MMF. The trace fluctuation in front of the vibration position is drastic and the trace at the vibration position and each position behind it is quite smooth. So, the obvious contrast in the OTDR trace benefits the vibration event location. As the trace averaging method takes into account the time duration and accumulation process of the vibration signal, we infer that it can overcome the error caused by the randomness of the sensing signal (the backscattering Rayleigh light), which is a big problem in the successive difference or trace subtraction method.

However, in the generally used trace subtraction method, the vibration events cause change in the optical polarization state and phase at the vibration position and each position behind, and the optical polarization state in the other region along the fiber is considered to be stable or slowly changing. Then, by trace subtraction, the final OTDR trace fluctuates drastically at the vibration position and each position behind. As this method does not consider the time duration characteristics of vibration events, it is easy to cause a high false alarm rate due to the very high polarization sensitivity.

**Figure 5:**
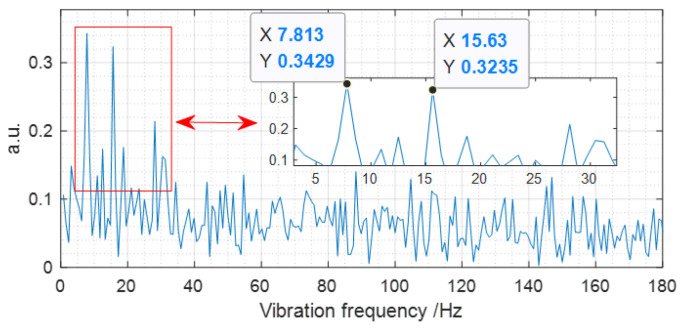
The spectrum of the vibration event loaded on MMF.

Once the vibration event is located, the next task is to extract the vibration spectrum. The FFT method is commonly used in DVS for multiple point vibration detection [22]. When the FFT algorithm is adopted to compute the data at the vibration position, we find that the vibration spectrum cannot be effectively extracted. So, we consider the averaging of the data of a certain position range to improve SNR of the vibration signal. Then, the data at the vibration position and positions within 20, 50, 100, 1000, and 5000 m and all the positions behind the vibration position are averaged, respectively. Finally, by FFT, the obtained spectrum is consistent with that shown in Figure 5. The measured vibration frequency is 7.8 Hz, which agrees with the event loaded on MMF well. In addition, there is a frequency doubling component in Figure 5, and it is inferred that this is caused by the fiber vibration interaction from different positions with the optical polarization state propagating in FUT.

**Figure 6:**
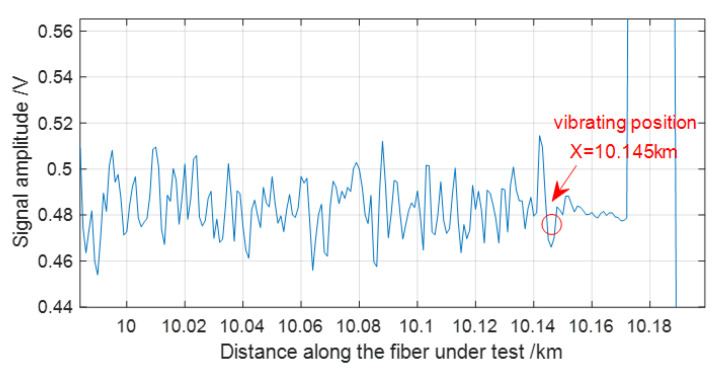
The averaged OTDR trace with a vibration event at 10.145 km.

**Figure 7:**
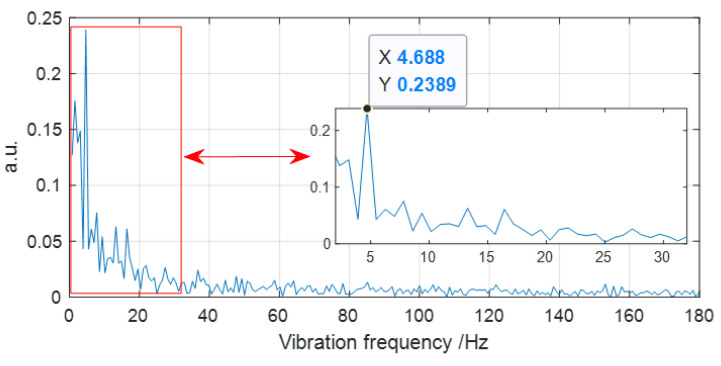
The spectrum of the vibration event loaded on MMF at 10.145 km.

In addition, we also load the vibration event at the fiber end at the position at 10.145 km, and 640 OTDR traces are also obtained with an average number of 10 times. Then, the data of the 640 OTDR traces are averaged again and the result is shown in Figure 6. It can be observed in Figure 6 that there is a very obvious difference between the vibration region and the non-vibration region. The vibration event is at the beginning of the rapid convergence region of the trace. In the same way, by averaging the data at the vibration position and the positions behind it and using the FFT algorithm, the vibration spectrum is extracted as shown in Figure 7. The vibration frequency also agrees well with the event loaded on FUT. The experimental results indicate that it is a very effective method for averaging data within a certain position range, which helps improve SNR of the vibration signal. In a polarization-sensitive OTDR system with SMF, the data at a certain position is directly analyzed by FFT and the vibration spectrum can be accurately extracted [22]. However, in our experiment with MMF, the data within a certain position range have to be averaged before spectrum analysis by FFT. Therefore, we infer that the randomness of the optical polarization state and the phase of the Rayleigh light in MMF has a great influence on the vibration signal, so data averaging within a certain position range is essential. This is a big difference in comparison with the method used for conventional polarization-sensitive OTDR using SMF.

## 4. Conclusions

Based on the polarization-sensitive OTDR system structure, a distributed vibration sensing system using MMF was proposed and experimentally demonstrated. The vibration event is considered to be a process that diversifies the polarization state and phase of Rayleigh scattering light, so that it can rapidly reduce fading noise in the OTDR trace. Therefore, the OTDR trace averaging method was adopted for fading noise reduction. It can also balance the data volume and data processing time for low-frequency vibration event analysis. As the randomness of Rayleigh scattering signals can be greatly suppressed by vibration events, the proposed DVS system has a lower false alarm rate. In the experiment, by trace averaging, the OTDR trace behind the position of the vibration event rapidly converged and the vibrating and non-vibrating regions in the OTDR trace were extremely obvious. Thus, this provides a good method for locating vibration events. In comparison with the trace subtraction method, in locating the vibration event, the trace average process improves the measurement SNR to ensure the accuracy. However, it negatively affects the dynamic measurement performance, such as the frequency response range. Then, a method that averages the data at the vibration position and a certain position range behind was proposed to improve SNR of the vibration signal. Using the FFT algorithm, the vibration spectrum was accurately extracted. For vibration spectrum extraction, this method saves time compared with the method that first analyzes the spectrum of each position along FUT and then locates the vibration events. Because the vibration event is located first, the spectrum extraction for vibration events is more targeted. The proposed DVS can be used for the vibration monitoring and event warning of oil pipelines, power cables, etc.

## Data Availability

Not applicable.
